# Magnesium Metabolism in Chronic Alcohol-Use Disorder: Meta-Analysis and Systematic Review

**DOI:** 10.3390/nu13061959

**Published:** 2021-06-07

**Authors:** Flora O. Vanoni, Gregorio P. Milani, Carlo Agostoni, Giorgio Treglia, Pietro B. Faré, Pietro Camozzi, Sebastiano A. G. Lava, Mario G. Bianchetti, Simone Janett

**Affiliations:** 1 Family Medicine Institute, Faculty of Biomedical Science, Università della Svizzera Italiana, 6900 Lugano, Switzerland; flora.vanoni@hotmail.com (F.O.V.); mario.bianchetti@usi.ch (M.G.B.); 2 Pediatric Unit, Fondazione IRCCS Ca’ Granda Ospedale Maggiore Policlinico, 20122 Milan, Italy; carlo.agostoni@unimi.it; 3 Department of Clinical Sciences and Community Health, Università degli Studi di Milano, 20122 Milan, Italy; 4 Pediatric Institute of Southern Switzerland, Ospedale San Giovanni, 6500 Bellinzona, Switzerland; 5 Academic Education, Research and Innovation Area, General Directorate, Ente Ospedaliero Cantonale, 6500 Bellinzona, Switzerland; giorgio.treglia@eoc.ch; 6 Faculty of Biomedical Science, Università della Svizzera Italiana, 6900 Lugano, Switzerland; 7 Department of Internal Medicine, Ente Ospedaliero Cantonale, 6600 Locarno, Switzerland; pietroBenedetto.Fare@eoc.ch; 8 Department of Internal Medicine, Ente Ospedaliero Cantonale, 6500 Bellinzona, Switzerland; pietro.camozzi@eoc.ch (P.C.); Simone.Janett@eoc.ch (S.J.); 9 Pediatric Cardiology Unit, Department of Pediatrics, Centre Hospitalier Universitaire Vaudois, University of Lausanne, 1011 Lausanne, Switzerland; webmaster@sebastianolava.ch

**Keywords:** magnesium, hypomagnesemia, depletion, kidney, diet, alcohol-use, electrolytes

## Abstract

Chronic alcohol-use disorder has been imputed as a possible cause of dietary magnesium depletion. The purpose of this study was to assess the prevalence of hypomagnesemia in chronic alcohol-use disorder, and to provide information on intracellular magnesium and on its renal handling. We carried out a structured literature search up to November 2020, which returned 2719 potentially relevant records. After excluding non-significant records, 25 were retained for the final analysis. The meta-analysis disclosed that both total and ionized circulating magnesium are markedly reduced in chronic alcohol-use disorder. The funnel plot and the Egger’s test did not disclose significant publication bias. The I^2^-test demonstrated significant statistical heterogeneity between studies. We also found that the skeletal muscle magnesium content is reduced and the kidney’s normal response to hypomagnesemia is blunted. In conclusion, magnesium depletion is common in chronic alcohol-use disorder. Furthermore, the kidney plays a crucial role in the development of magnesium depletion.

## 1. Introduction

Chronic alcohol-use disorder is a frequent, disabling condition [1], which is often associated with electrolyte derangements such as metabolic acidosis or alkalosis, hypokalemia, hyponatremia, hypocalcemia, and hypophosphatemia [2]. Magnesium depletion has also been reported [2].

Magnesium balance is a function of intake, distribution between the extra- and the intracellular compartments, and excretion. Approximately one-third is absorbed in the small bowel. In the extracellular fluid, magnesium is ionized and bound to either small anions or proteins, primarily albumin. However, most of the body’s magnesium is inside cells, bound to adenosine triphosphate and other intracellular nucleotides and enzymes, and an integral component of bone mineral. The renal handling of magnesium differs from that of other ions because the major sites of transport are the loop of Henle and the distal convoluted tubule [3,4].

There is a need to summarize the knowledge about the metabolism of magnesium in chronic alcohol-use disorder. Hence, we reviewed the literature addressing the magnesium balance in this condition. The purposes of our study were to assess the intake, the intestinal metabolism, the extra- and intracellular levels, and the kidney handling of magnesium in this condition.

## 2. Materials and Methods

### 2.1. Data Sources and Searches

This review was accomplished following the Preferred Reporting Items for Systematic Reviews and Meta-Analyses recommendations [5]. We carried out a structured [6] literature search up to November 2020 with no date and language limits in the databases of the United States National Library of Medicine, Excerpta Medica, and Web of Science. We also reviewed reference lists of retained articles and our personal files. The search strategy incorporated following terms: (“magnesium” OR “ionized magnesium” OR “intracellular magnesium” OR “hypomagnesemia” OR “magnesium depletion” OR “magnesium deficiency” OR “dietary magnesium” OR “intestinal magnesium absorption”) AND (“alcohol” OR “alcohol-use disorder” OR “alcoholism” OR “withdrawal”).

### 2.2. Selection Criteria

We retained full-length, observational reports addressing the prevalence of hypomagnesemia, either total or ionized, in subjects affected by chronic alcohol-use disorder. Of interest were reports including 5 or more chronic alcohol-use disorder subjects who were investigated with respect to their blood magnesium concentration. Studies addressing the intracellular magnesium concentration and the kidney magnesium handling were also included. Chronic alcohol-use disorder subjects in treatment with drugs with a potential to cause hypomagnesemia such as aminoglycosides, calcineurin inhibitors, diuretics, or proton-pump inhibitors were excluded [4,7,8].

### 2.3. Definitions

The diagnosis of chronic alcohol-use disorder made in the original reports was retained. The kidney normally reduces the magnesium excretion to very low levels following depletion of this ion [4,9]. Consequently, in subjects with hypomagnesemia and normal blood creatinine, a 24-h magnesium excretion greater than 500 µmol or a fractional magnesium clearance above 2.0 × 10^−2^ indicates renal magnesium wasting [4,9].

### 2.4. Data Extraction

All data were extracted using a pilot-tested sheet. We extracted the following data from the retrieved studies that were used for the pooled analyses: number of patients with chronic alcohol-use disorder and healthy subjects; mean and standard deviation of total and ionized circulating magnesium level in patients with chronic alcohol-use disorder and healthy subjects; number of cases of total hypomagnesemia in patients with chronic alcohol-use disorder; laboratory technique.

### 2.5. Analysis

Results are expressed as mean ± SD or as frequency. The two-tailed t-test was used for non-pooled analysis. Significance was set at *p* < 0.05.

We conducted three different pooled analyses: first, an analysis of observational studies investigating mean difference of total magnesium level in patients with chronic alcohol-use disorder compared to healthy subjects; second, an analysis of observational studies investigating mean difference of ionized circulating magnesium level in patients with chronic alcohol-use disorder compared to healthy subjects; third, an analysis of the prevalence of total hypomagnesemia in chronic alcohol-use disorder. The comparison of mean total and ionized circulating magnesium level in patients with chronic alcohol-use disorder and healthy subjects was expressed as mean difference (MD) from the retrieved articles and a pooled MD was calculated for these parameters. The prevalence of total hypomagnesemia in chronic alcohol-use disorder was calculated from the retrieved studies and a pooled prevalence was calculated through a meta-analysis. A random-effects model was used for statistical pooling of data. Pooled data represented weighted mean or proportion, which were related to the sample size of the individual studies. Pooled results were presented with 95% confidence intervals (95%-CI) and displayed using forest plots.

An I^2^-test was also performed to test for heterogeneity between studies; this test describes the percentage of variation across studies that is due to heterogeneity rather than chance. The presence of significant heterogeneity was defined as an I-square value of more than 50%. For publication bias, evaluation funnel plots and Egger’s test were used when a sufficient number of studies was available. Statistical analyses were performed using StatsDirect statistical software (StatsDirect Ltd., Birkenhead, UK).

## 3. Results

### 3.1. Search Results

**Figure 1:**
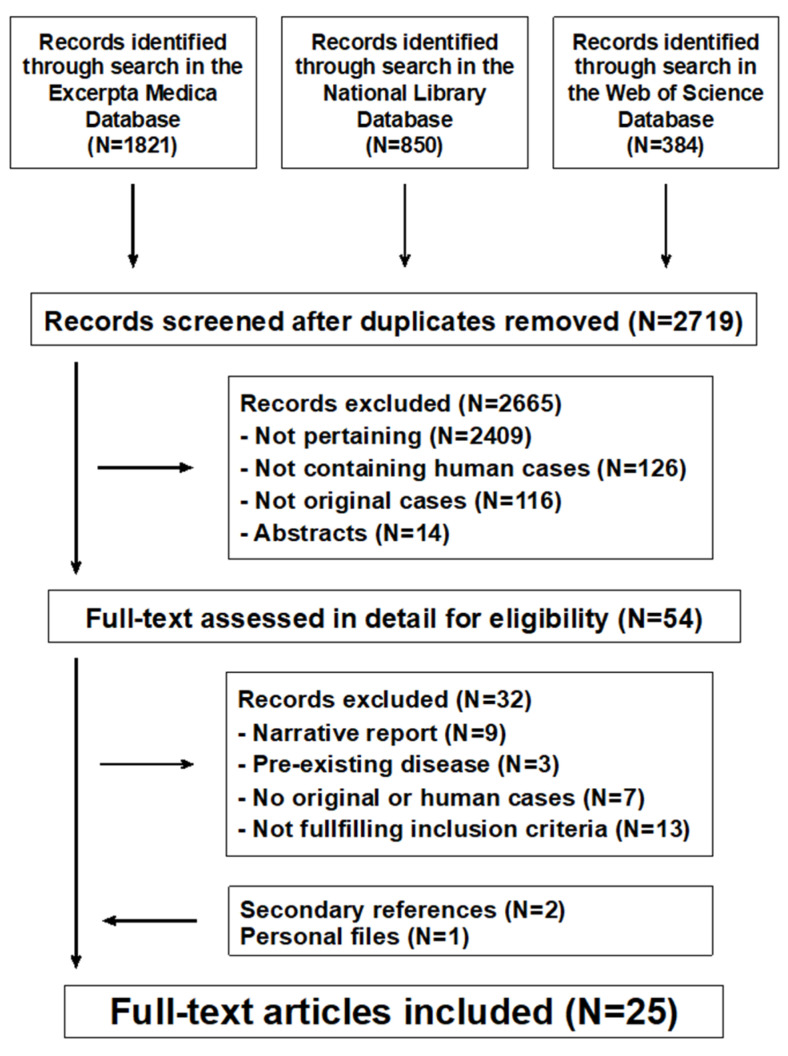
Magnesium metabolism in chronic alcohol-use disorder. Flowchart of the literature search process.

The literature search returned 2719 potentially relevant records (Figure 1). After excluding non-significant records, 25 potentially eligible reports were considered [10,11,12,13,14,15,16,17,18,19,20,21,22,23,24,25,26,27,28,29,30,31,32,33,34]: 13 from the United States of America, 2 from Greece, 2 from Italy, and one each from Croatia, Denmark, Finland, Israel, Poland, Singapore, Sweden, and the United Kingdom. All reports were written in English. No reports about dietary intake or intestinal metabolism of magnesium were identified.

### 3.2. Extracellular Magnesium Concentration

#### 3.2.1. Total Magnesium

Thirteen reports compared the total circulating magnesium level in 546 subjects affected by chronic alcohol-use disorder and 660 healthy controls [11,12,15,21,22,23,25,28,29,30,32,33,34]. Magnesium was determined by colorimetry in eight and by atomic absorption spectrometry in the remaining five reports. Circulating magnesium was found to be significantly reduced in nine of the mentioned studies [11,12,15,21,22,23,25,29,32].

On the other hand, 12 reports [10,13,14,16,17,18,19,20,24,26,27,31] addressed the prevalence of hypomagnesemia in 538 subjects with chronic alcohol-use disorder. Colorimetry was employed in nine and atomic absorption spectrometry in three reports. Hypomagnesemia was observed in 199 (27%) of the patients.

#### 3.2.2. Ionized Magnesium

Three reports [29,32,34] determined by direct potentiometry the ionized magnesium concentration in 171 subjects with alcohol-use disorder and 165 healthy controls. A tendency to ionized hypomagnesemia was noted in all the mentioned reports.

#### 3.2.3. Pooled Analyses

**Figure 2:**
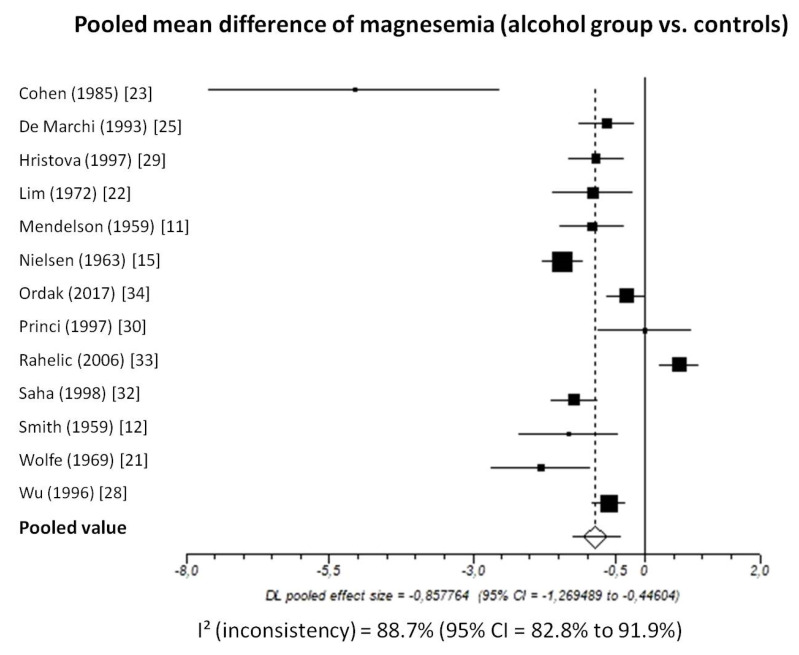
Forest plot of individual studies and pooled mean difference of magnesemia between alcohol group and controls, including 95% confidence intervals (95%-CI). The size of the squares is related to the weight of each study. Number of patients and control subjects in each study: Cohen [23]: controls *n* = 5, patients *n* = 5; De Marchi [25]: controls *n* = 42, patients *n* = 30; Hristova [29]: controls *n* = 40, patients *n* = 31; Lim [22]: controls *n* = 87, patients *n* = 9; Mendelson [11]: controls *n* = 18, patients *n* = 50; Nielsen [15]: controls *n* = 157, patients *n* = 48; Ordak [34]: controls *n* = 50, patients *n* = 100; Princi [30]: controls *n* = 14, patients *n* = 10; Rahelic [33]: controls *n* = 50, patients *n* = 105; Saha [32]: controls *n* = 75, patients *n* = 40; Smith [12]: controls *n* = 13, patients *n* = 12; Wolfe [21]: controls *n* = 12, patients *n* = 18; Wu [28]: controls *n* = 97, patients *n* = 88.

Pooled MD of total magnesium level in patients with chronic alcohol-use disorder compared to healthy subjects, taking into account 13 studies (1206 patients, 546 with chronic alcohol-use disorder and 660 healthy subjects), was −0.86 (95%-CI: −1.27 to −0.45) mmol/L (Figure 2).

**Figure 3:**
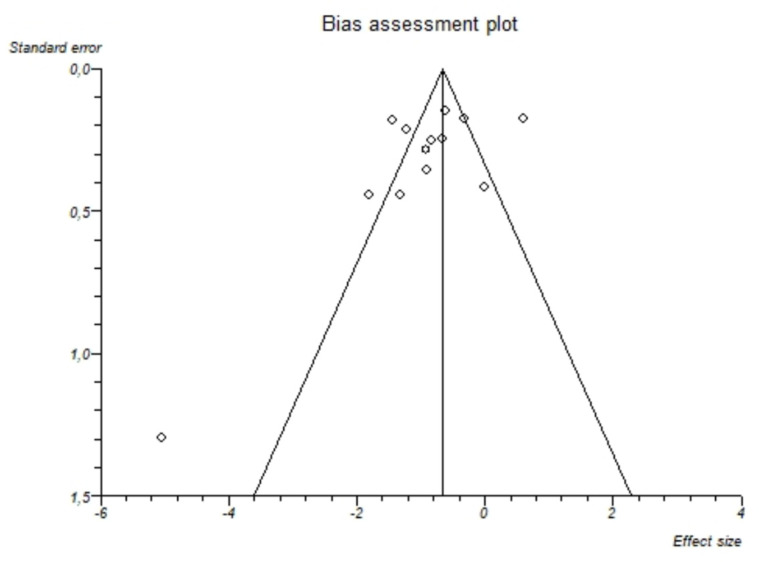
Bias assessment plot (funnel plot) about the pooled mean difference of magnesemia between alcohol group and controls. The visual analysis does not show a significant asymmetry and the presence of a publication bias is not demonstrated.

I^2^-test was 88.7% (95%-CI: 82.8–91.9) demonstrating significant statistical heterogeneity between studies. No significant publication bias was found through the visual analysis of funnel plot (Figure 3) and Egger’s test (*p* = 0.15).

Pooled mean difference of total magnesium level in patients with alcohol-use disorder compared to controls based on the different assays was as follows: atomic absorption spectrometry: −1.60 (95%-CI = −2.67 to −0.05); I^2^: 93%; colorimetry: −0.79 (95%-CI = −1.10 to −0.48); I^2^: 76%

**Figure 4:**
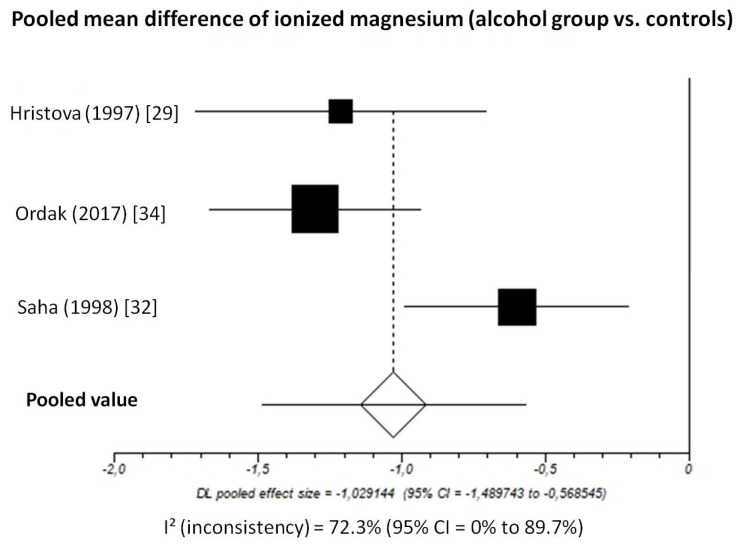
Forest plot of individual studies and pooled mean difference of ionized magnesium between alcohol group and controls, including 95% confidence intervals (95%-CI). The size of the squares is related to the weight of each study. Number of patients and control subjects in each study: Hristova [29]: controls *n* = 40, patients *n* = 31; Ordak [34]: controls *n* = 50, patients *n* = 100; Saha [32]: controls *n* = 75, patients *n* = 40.

Pooled MD of ionized magnesium level in patients with chronic alcohol-use disorder compared to healthy subjects, taking into account 3 studies (336 patients, 171 with chronic alcohol-use disorder and 165 healthy subjects), was −1.03 (95%-CI: −1.49 to −0.57) mmol/L (Figure 4).

The I^2^-test was 72.3% (95%-CI: 0–89.7%), demonstrating significant statistical heterogeneity between studies. No analysis of publication bias was performed due to the limited number of studies available for this analysis.

**Figure 5:**
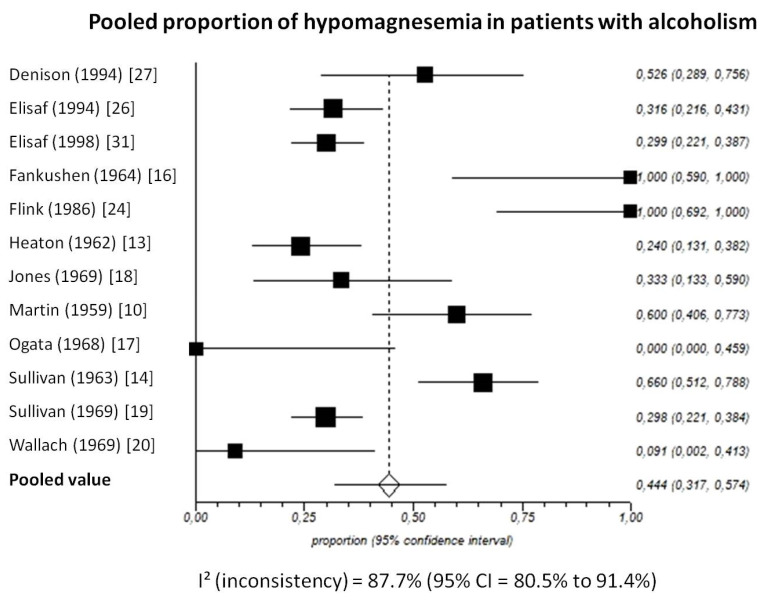
Forest plot of individual studies and pooled proportion of hypomagnesemia in patients with alcoholism, including 95% confidence intervals (95%-CI). The size of the squares is related to the weight of each study. Number of subjects in each study: Denison [27]: *n* = 19; Elisaf [26]: *n* = 79; Elisaf [31]: *n* = 127; Fankushen [16]: *n* = 7; Flink [24]: *n* = 10; Heaton [13]: *n* = 50; Jones [18]: *n* = 18; Martin [10]: *n* = 30; Ogata [17]: *n* = 6; Sullivan [14]: *n* = 50; Sullivan [19]: *n* = 131; Wallach [20]: *n* = 11.

Pooled prevalence of total hypomagnesemia in patients with chronic alcohol-use disorder, taking into account 12 studies (538 patients), was 44.4% (95%-CI: 31.7 to 57.4) (Figure 5).

**Figure 6:**
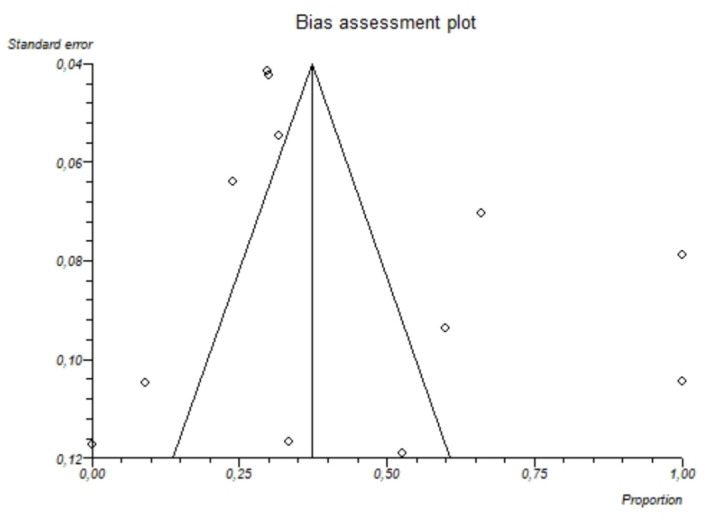
Bias assessment plot (funnel plot) showing the pooled proportion of hypomagnesemia in patients with alcoholism. The visual analysis does not show significant asymmetry and the presence of a publication bias is not demonstrated.

The I^2^-test was 87.7% (95%-CI: 80.5–91.4), demonstrating significant statistical heterogeneity between studies. No significant publication bias was found through the visual analysis of funnel plot (Figure 6) and Egger’s test (*p* = 0.32).

### 3.3. Intracellular Magnesium

#### 3.3.1. Intra-Erythrocytic Magnesium

The total red blood cell magnesium concentration was investigated in four reports. Two reports analyzed [13,33] the prevalence of low total intra-erythrocytic magnesium level in 69 patients and found a reduced value in just three (4.3%) cases. Two reports [12,34] compared total red blood cell magnesium in chronic alcohol-use disorder subjects and healthy controls. Smith et al. [12] found quite meaningfully reduced content in patients compared to controls (1.60 ± 0.38 mmol/L, *n* = 12, vs. 2.65 ± 0.17 mmol/L, *n* = 13, *p* < 0.0001).

More recently, Ordak et al. [34] assessed both total and ionized red blood cell magnesium. The total magnesium level was found to be similar in patients and controls (1.64 ± 0.80 mmol/L, *n* = 100, vs. 1.60 ± 0.70 mmol/L, *n* = 50, *p* = 0.764). By contrast, the ionized magnesium level was significantly lower in chronic alcohol-use disorder patients than in healthy controls (0.36 ± 0.10 mmol/L; *n* = 100, vs. 0.71 ± 0.14 mmol/L, *n* = 50, *p* < 0.0001).

#### 3.3.2. Intra-Lymphocytic Magnesium

The total intra-lymphocytic magnesium concentration was investigated in two small reports. Princi et al. [30] found almost identical (*p* = 0.799) intra-lymphocytic magnesium levels in chronic alcohol-use disorder patients (56.9 ± 22.4 nmol/mg protein; *n* = 10) and healthy controls (60.2 ± 35.8 nmol/mg protein; *n* = 14). Also Cohen et al. [23] found similar (*p* = 0.077) intralymphocytic levels in both groups: 36.6 ± 2.3 mmol/kg lymphocyte-dry weight (*n* = 5) versus 42.1 ± 5.6 mmol/kg lymphocyte-dry weight (*n* = 5).

#### 3.3.3. Bone Magnesium Content

Two small studies addressed the bone magnesium concentration [22,23]. Cohen et al. [23] found a slightly (by 17%) but significantly lower (*p* < 0.03) magnesium trabecular bone content in patients (175 ± 23 mmol/kg; *n* = 5) than in controls (209 ± 18 mmol/kg; *n* = 5). The cortical bone magnesium content, however, was found to be similar in the two groups (no quantitative data provided in the report). Lim et al. [22] included exclusively hypomagnesemic patients and found a slightly (by 9.1%) but significantly lower (*p* < 0.03) content in patients (101 ± 5 mmol/kg; *n* = 9) than in healthy controls (111 ± 12 mmol/kg; *n* = 47).

#### 3.3.4. Skeletal Muscle Magnesium Content

The magnesium concentration in skeletal muscle [18] was shown to be reduced (*p* < 0.001) by 16% in subjects with chronic alcohol-use disorder (30.6 ± 3.4 mmol/kg; *n* = 20) as compared with controls (36.3 ± 2.3 mmol/kg; *n* = 12). Similarly, Lim et al. [22] found a reduced skeletal muscle magnesium concentration in 9 out of 10 subjects with chronic alcohol-use disorder.

### 3.4. Kidney Magnesium Handling

Five reports investigated the kidneys’ magnesium handling in chronic alcohol-use disorder. Sullivan et al. [14] found a similar urinary magnesium excretion in chronic alcohol-use disorder patients with normal (*n* = 11) and reduced (*n* = 24) total circulating magnesium. De Marchi et al. [25] assessed the fractional renal magnesium clearance in a group of 61 chronic alcohol-use disorders patients with a tendency towards hypomagnesemia and 42 healthy controls. This value was significantly higher (*p* < 0.0001) in patients (3.3 ± 1.8 × 10^−2^) than in controls (1.9 ± 1.2 × 10^−2^). In the 18 patients with hypomagnesemia, the latter authors found a fractional magnesium clearance above 2.0 × 10^−2^, i.e., features consistent with renal magnesium wasting. Three further studies [16,20,26] investigated a total of 42 chronic alcohol-use disorder patients with hypomagnesemia and found in all cases a 24-h urinary magnesium excretion of more than 0.5 mmol, i.e., features consistent with renal magnesium wasting.

## 4. Discussion

Magnesium plays an essential role in the physiology of the brain, heart, and skeletal muscles; has anti-inflammatory properties; and acts as a calcium-antagonist [3,4]. The results of this meta-analysis and systematic review on magnesium metabolism in chronic alcohol-use disorder may be summarized in three points: (1) both total and ionized circulating magnesium are markedly reduced; (2) skeletal muscle magnesium content is also reduced; (3) the normal response to hypomagnesemia of the kidney is blunted.

Circulating magnesium exists in the ionized state, the most interesting with respect to physiological properties, and in the undissociated form [3,4,35]. Since, in chronic alcohol-use disorder, hypoalbuminemia is common [36], it has been speculated that in this condition low total circulating magnesium is spurious and brought about by coexisting hypoalbuminemia. Our study demonstrates that, in chronic alcohol-use disorder, hypomagnesemia is not spurious.

The total body magnesium content is approximately 1000 mmol. Approximately 1% is in the extracellular fluid compartment, 60–65% is found in bone, 20% is localized in the muscle, and the remainder is found in other tissues [3,4]. Our analysis indicates that hypomagnesemia is habitually linked with skeletal muscle magnesium depletion (in these cells magnesium is vital for glycolysis, formation of cyclic adenosine monophosphate, and reactions that use or produce energy). On the other hand, the results addressing the intra-erythrocytic, intra-lymphocytic, and bone magnesium values appear to be rather inconsistent.

The kidney plays a crucial role in magnesium homeostasis and one can distinguish hypomagnesemia due to poor dietary intake or impaired intestinal metabolism from depletion caused by kidney wasting by measuring the 24-h urinary excretion or the fractional clearance of this ion [4,9]. The present review documents the presence of inappropriately high magnesium excretion in hypomagnesemic patients with chronic alcohol-use disorder. It is therefore concluded that an impaired kidney magnesium handling plays a critical role in the development of magnesium deficiency.

The most relevant strength of the study relates to the comprehensive and exhaustive analysis, which aimed at surveying the entirety of literature on this issue. Furthermore, we did not find a significant publication bias in the pooled analyses. The limitations of the available literature on magnesium metabolism in chronic alcohol-use disorder are the failure to clearly define what is meant by this condition, the inexistent documentation of nutritional status [37,38], the absence of studies addressing the dietary intake and the intestinal metabolism of magnesium [37,38], the paucity of data on common comorbidities such as liver disease or pancreatitis [39,40], and the likely inaccurate evaluation of the effect of drug medication [7].

Bearing in mind the mentioned limitations, future research should take into consideration the following seven points:

1. Definition of alcohol-use disorder: The DSM-5 criteria for alcohol-use disorders, its severity and its various subtypes should be used to enroll patients in future investigations.
2. Nutritional status: Nutritional status is often impaired in alcohol-use disorder [37,38]. In fact, affected subjects often ingest too little of essential nutrients. Furthermore, alcohol may prevent the body from properly absorbing and using nutrients [37,38]. The determination of mid-arm muscle circumference, triceps skinfold thickness, mid-arm circumference, and subscapular skinfold thickness is imperative for the clinical evaluation of nutritional status. The gold standard for the diagnosis of poor intestinal absorption is the determination for fat on 72-h stool collection. Near-infrared analysis is superior because it allows for simultaneous measurement of fecal fat, nitrogen, and carbohydrates in a single sample. Finally, stable isotopes represent a useful technique for determining intestinal absorption of magnesium [41].
3. Dietary magnesium intake: The strategy to collect information on dietary magnesium intake is nowadays an image-assisted dietary food record [42].
4. Liver disease: Alcohol-associated liver disease might play a role in modulating the severity of magnesium depletion [39]. Magnesium metabolism should be investigated separately in patients without and with liver diseases.
5. Pancreatitis: Attacks of pancreatitis are common in chronic alcohol-use disorder. Since hypomagnesemia can occur in this condition, we advise the determination of lipase in future studies [40].
6. Drug-induced magnesium depletion: The reports selected for this review did not include subjects with drug-induced hypomagnesemia. Since an association between hypomagnesemia and proton pump inhibitors was first suspected in 2006 [7], we speculate that these agents were sometimes not specifically imputed as a cause of hypomagnesemia. As a consequence, the possible role of drug-induced hypomagnesemia should be carefully addressed in future studies.
7. Magnesium status: Ideally, total circulating magnesium level should be measured by atomic absorption spectrometry. It is true, however, that many colorimetric methods have been validated. Potentiometric sensors are employed for the measurement of blood-ionized magnesium. Since the selectivity of these sensors for magnesium over calcium is unsatisfactory, the results are adjusted for calcium interference. It has been suggested that magnesium content in red blood cells, peripheral lymphocytes, bones, and skeletal muscles might be an index of magnesium status. However, the available evidence does not support the use of intracellular magnesium levels as an indicator of overall bodily magnesium status [3,4,43].

## 5. Management

No study has so far investigated the management of magnesium depletion in chronic alcohol-use disorder. Hypomagnesemia is mostly mild and presents without or minimal symptoms [2]. In this setting, avoidance of drugs with a potential to induce hypomagnesemia (e.g., the substitution of proton-pump inhibitors with histamine type-2 receptor antagonists respectively of thiazides with potassium-sparing diuretics) is advised. Owing to the effects of alcohol on kidney tubular function, replacement is not expected to be very effective, as in inherited disorders of renal magnesium handling [4]. The restoration of tubular function and magnesium status occurs approximately 4 weeks after alcohol abstinence [25]. After intravenous administration, only a small portion of magnesium is retained because most is excreted in the urine [4,25]. For this reason, repeated oral dosing may be preferred to repair the deficit. Intravenous replacement is required for symptomatic cardiac arrhythmias such as torsade de pointes or neuromuscular irritability [2]. The prescription of magnesium for alcohol withdrawal including delirium tremens has been debated. However, there is so far insufficient evidence to prescribe magnesium in this setting [44].

Future studies are warranted to explore the risk stratification of magnesium depletion and the intracellular magnesium metabolism in chronic alcohol-use disorder.

Magnesium is deemed to be the fifth but forgotten electrolyte [3]. In conclusion, this review demonstrates that magnesium depletion is common in chronic alcohol-use disorder. Contrary to what common sense would suggest, the kidney plays a crucial role in the development of magnesium depletion. We urge that patients with chronic alcohol-use disorder have frequently blood magnesium measured. This suggestion is supported by recent data that implicate magnesium depletion in high mortality in this condition [45,46].

## Data Availability

Data are available upon the corresponding author after reasonable request.
